# Comparison of Biochemical and Hematological Profiles in Patients of Extrapulmonary and Pulmonary Tuberculosis at a Tertiary Care Center

**DOI:** 10.7759/cureus.35778

**Published:** 2023-03-05

**Authors:** Shyama Shyama, Vishnu S Ojha, Ratnadeep Biswas, Luv Luv, Gurleen Kaur, Yash Jaiswal, Asiff N Aneef

**Affiliations:** 1 Internal Medicine, All India Institute of Medical Sciences, Patna, IND

**Keywords:** cross-sectional studies, hematologic tests, biomarkers, adult, pediatrics, extrapulmonary tuberculosis, pulmonary tuberculosis, tuberculosis

## Abstract

Background

Tuberculosis (TB) is a disease of global concern, especially in countries like India. Pulmonary TB (PTB) and extrapulmonary TB (EPTB) differ a lot when it comes to presentations, treatment, and outcomes. The biochemical and hematological test can serve as a marker reflecting the response to treatment in various types of TB, resulting in a better prognosis. Therefore, this study was conducted to compare the biochemical and hematological profiles in patients of extrapulmonary and pulmonary tuberculosis in adults and children.

Methods

TB cases were divided into four categories: PTB adult, EPTB adult, PTB pediatrics, and EPTB pediatrics. Forty-nine patients in each category were selected, resulting in a total of 196 patients. The sample size was met via convenience sampling. A total of 27 parameters were compared. Mann-Whitney U tests were used for statistical analysis.

Results

It was observed that serum calcium levels in PTB cases (11.65, 1.15; median and inter-quartile range (IQR), respectively) were significantly different from those in EPTB cases (9.18, 1.03; p<0.001). The median serum sodium levels in EPTB cases (139.49, 6.86) were higher than in PTB cases (130.10, 5.77; p<0.001). For total platelet count levels, a significant difference was observed between PTB (337.00, 180.75) and EPTB cases (278, 159.25; p=0.006). In EPTB cases, the total red blood count (RBC) count levels (4.47, 0.96) were higher than in PTB cases (4.24, 0.89; p=0.036). Biochemical and hematological parameters between pediatrics and adult age groups were compared, and it was observed that the median values (IQR) of serum phosphorus, total white blood cells (WBC), and platelet count in pediatric cases were 5.16 (1.09), 14.75 (6.03), and 350.00 (155.75), respectively, and were higher from those in adult cases 3.78 (0.97); 8.35 (6.66) and 264 (181.5), respectively (p<0.001). For serum creatinine levels, a significant rise was observed between PTB 0.54 (0.19) and EPTB cases 0.57 (0.16) (p<0.001). It was also observed that alanine transaminase (ALT) levels were higher in adults (18.90 (17.83)) than in the pediatric age group (24.70 (28.67); p=0.042) while alkaline phosphatase (ALP) was higher in the pediatric age group (108.95 (78.37)) than in adults (94.25 (47.92); p=0.003).

Conclusion

Serum calcium levels and total WBC counts were higher in PTB cases, while the levels of serum sodium and total RBC counts were higher in EPTB cases. ALT, serum phosphorus, total WBC counts, and total platelet counts were higher in the pediatric age group, while ALP, serum urea, and creatinine levels were higher in adults. Increased tissue damage and severity of disease in the pediatric age group, reactive thrombocytosis due to biogenesis in lungs, and abnormal anti-diuretic hormone secretion in PTB cases may be possible explanations for these findings. These findings may help clinicians in the early identification of potential complications, and further studies on these parameters should be conducted.

## Introduction

Tuberculosis (TB) has always been a global concern because of its high morbidity and mortality, especially in developing countries and, more so, in countries like India. The incidence of tuberculosis ranges from 9 to 11 million people worldwide, comprising 5.8 million men, 3.2 million women, and nearly a million children [1]. Mycobacterium tuberculosis causes the airborne disease TB, which typically affects the lungs and results in severe coughing, fever, and chest pains [2]. TB is contracted primarily through droplets. It is an alveolar macrophage prototypic intracellular pathogen that has expertly adapted to infect humans. Alveolar macrophages are the main carriers of infection and sickness [3]. Thus, it causes a chronic granulomatous disease that affects the lungs and is known as pulmonary tuberculosis (PTB), and in the case tuberculosis is diagnosed bacteriologically in other parts of the body, i.e., apart from the lungs, such as the abdomen, lymph nodes, meninges or genitalia, it is referred to as extrapulmonary tuberculosis (EPTB) [4]. Although they share a common pathogen, PTB and EPTB differ a lot when it comes to presentations, treatment, and outcomes. A lot of literature exists about tuberculosis, both pulmonary and extrapulmonary, but not a lot of studies have been done comparing the two. We found one that reported early changes in plasma concentration of inflammatory markers comparing PTB and EPTB cases [5].

As TB is an endemic disease in India, it is likely that by the time a person reaches adulthood, he may already have a latent infection of TB. And since it is already reported that latent TB infections have a protective effect on subsequent infections while, at the same time, there is a faster progression in previously treated patients of TB [6]. Thus, age also becomes a key determinant that needs to be evaluated.

Despite numerous studies, laws, policies, and programs like National Tuberculosis Elimination Program (NTEP) and despite having anti-tubercular therapy (ATT) regimes that are quite effective, the burden of tuberculosis is reducing very slowly, and the management of tuberculosis remains highly variable throughout the world and in the country, especially in the pediatric age group and in EPTB cases. This may be attributed to a lack of timely diagnosis, variable prognostic evaluation, late identification of complications, and a general lack of knowledge.

The biochemical and hematological test can serve as a marker reflecting the response to treatment in various types of tuberculosis, resulting in a better prognosis [7]. Therefore, this study was conducted to compare the biochemical and hematological profiles of patients of extrapulmonary and pulmonary tuberculosis in adults and children in the departments of general medicine, pulmonary medicine, and pediatrics of All India Institute of Medical Sciences, Patna.

## Materials and methods

Ethical consideration

The research project was initiated after obtaining approval from the Institute Ethics Committee. Informed consent was obtained from all the subjects. Strict confidentiality was maintained with patient data throughout the study.

Sample size

Previously published literature by Morris et al. reported various deranged hematological and biochemical parameters among tuberculosis patients in the range of 17% to 80% and 43% to 94%, respectively [7]. Taking these reported prevalences into consideration for sample size, 7% absolute error, and 95% confidence interval. The largest sample size was calculated as 196 (with a 52% proportion of monocytopenia and elevated platelet count). Sample size calculation was done using an online calculator for the estimation of a single proportion [8], the results of which can be seen in Table 1.

**Table 1:** Sample size calculation

The proportion of deranged hematological or biochemical parameters among tuberculosis patients	Absolute error	Calculated sample size
Anaemia (60%)	7%	189
Leucocytosis (40%)	7%	189
Lymphopenia (17%)	7%	111
Elevated erythrocyte sedimentation rate (80%)	7%	126
Hypoalbuminemia (72%)	7%	159
Vitamin B12 (57%)	7%	193
Ferritin (94%)	7%	45
Hyponatremia (43%)	7%	193
Monocytopenia (52%)	7%	196
Elevated platelet count (52%)	7%	196

The total sample size included for evaluation (N=196) was divided into four equal categories: PTB adults, PTB pediatrics, EPTB adults, and EPTB pediatrics, with 49 patients in each category.

Study population

Patients microbiologically diagnosed with pulmonary and/or extrapulmonary tuberculosis were included in the study.

Inclusion and Exclusion Criteria

Patients of 16 years or below were considered in the pediatric age group, and those above 16 years were considered in the adult age group. Patients with microbiologically confirmed tuberculosis in different parts of the body other than the lung, such as the lymph nodes, meninges, or genitals, were classified as extrapulmonary tuberculosis (EPTB). Patients suffering from any other diseases or comorbidities were excluded based on history and physical examination.

Data collection

This hospital-based cross-sectional study was conducted over a period of six months. Individuals meeting the inclusion criteria were selected through convenient sampling. The results of their routine blood tests (including complete blood counts, liver function tests, and kidney function tests) before the initiation of ATT were recorded.

Statistical analysis

The data was cleaned and coded in Microsoft Excel (Microsoft, Redmond, Washington) and then it was analyzed using SPSS version 20.0 (IBM Inc., Armonk, New York) and Jamovi Software. All the parameters were checked for normality using the Shapiro-Wilk's test and Q-Q plots, and it was observed that most of the variables had a non-normal distribution (p<0.05). Therefore, Mann-Whitney U tests for two independent samples were used to compare median values of various parameters in two separate analyses; first considering the type of TB (PTB or EPTB), and second considering the age of patients (adults or children). Median values and inter-quartile ranges (IQR) were reported for the various lab parameters. Significance was attributed to a p-value less than 0.05.

## Results

On comparing the various biochemical and hematological parameters between all PTB cases (n_PTB_=98) and all EPTB cases (n_EPTB_=98) (including both adult and pediatric age groups) using the Mann-Whitney U test, it was observed that serum calcium levels in PTB cases (11.65, 1.15; median and IQR, respectively) were significantly different from those in EPTB cases (9.18, 1.03; p<0.001). The median serum sodium levels in EPTB cases (139.49, 6.86) were higher than in PTB cases (130.10, 5.77; p<0.001). For total platelet count levels, a significant difference was observed between PTB (337.00, 180.75) and EPTB cases (278, 159.25; p=0.006). In EPTB cases, the total red blood cell (RBC) counts (4.47, 0.96) were higher than in PTB cases (4.24, 0.89; p=0.036), as mentioned in Table 2.

**Table 2:** Comparison of parameters between pulmonary and extrapulmonary tuberculosis (n=98 in both groups) IQR - inter-quartile range *includes both pediatric and adult age groups The p-values in bold letters are statistically significant (<0.05)

Parameters	Pulmonary tuberculosis *		Extrapulmonary tuberculosis*		p-value
	Median	IQR	Median	IQR	
Total bilirubin	0.48	0.37	0.52	0.36	0.93
Direct bilirubin	0.13	0.13	0.15	0.12	0.215
Indirect bilirubin	0.37	0.23	0.37	0.25	0.491
Alanine transaminase	22.1	23.42	20.7	19.5	0.656
Aspartate transaminase	35.05	24.07	30.3	27.5	0.168
Alkaline phosphatase	97	64.5	99.75	57.13	0.737
Total protein	7.18	1.44	7.42	1.39	0.281
Albumin	3.57	0.72	3.53	0.77	0.911
Globulin	3.59	0.94	3.66	1.08	0.86
Albumin/globulin ratio	1	0.34	0.99	0.38	0.945
Serum urea	20.2	9.62	23.3	13.57	0.202
Serum creatinine	0.61	0.26	0.62	0.2	0.199
Serum uric acid	4.52	2.27	4.34	2.46	0.584
Serum calcium	11.65	1.15	9.18	1.03	<0.001
Serum phosphorus	4.22	1.74	4.48	1.39	0.381
Serum sodium	130.1	5.77	139.49	6.86	<0.001
Serum potassium	4.25	0.67	4.29	0.57	0.64
Serum chloride	99.04	6.26	99.3	6.44	0.453
Total white blood cell count	12.01	7.49	11.31	7.1	0.392
Total platelet count	337	180.75	278	159.25	0.006
Total red blood cell count	4.24	0.89	4.47	0.96	0.036
Total neutrophil count	78.5	16.52	75.35	13.22	0.447
Total lymphocyte count	16.8	12.02	18.8	11.1	0.107
Total monocyte count	3.1	1.7	3	1.6	0.378
Total eosinophil count	0.95	2.82	0.9	1.8	0.736
Total basophil count	0.3	0.3	0.29	0.3	0.215
Mean platelet volume	11.8	1.32	11.8	1.7	0.522

Biochemical and hematological parameters between all pediatric patients (n_p__ediatrics_=98) and all adult patients (n_a__dults_=98), including both PTB and EPTB cases, were compared, and it was observed that median values (IQR) of serum phosphorus, total white blood cells (WBC), and total platelet counts in pediatric cases were 5.16 (1.09), 14.75 (6.03), and 350.00 (155.75), respectively, and were higher from those in adult cases 3.78 (0.97), 8.35 (6.66), and 264 (181.5), respectively (p<0.001). For serum creatinine and urea levels, a significant rise was observed in PTB cases (0.54 (0.19)) and (20.45 (9.65)) when compared to EPTB cases (0.57 (0.16)) and (24.95(16.22); p<0.001 and p=0.006, respectively). It was also observed that alanine transaminase (ALT) levels were higher in adults (18.90 (17.83)) than in the pediatric age group (24.70 (28.67); p=0.042) while alkaline phosphatase (ALP) was higher in the pediatric age group (108.95 (78.37)) than in adults (94.25 (47.92); p=0.003), as mentioned in Table 3.

**Table 3:** Comparison of parameters between pediatric and adult age groups (n=98 in both groups) IQR - inter-quartile range *includes cases of both pulmonary and extrapulmonary tuberculosis The p-values in bold letters are statistically significant (<0.05)

Parameters	Pediatric age group*		Adult age group*		p-value
	Median	IQR	Median	IQR	
Total bilirubin	0.48	0.37	0.5	0.38	0.703
Direct bilirubin	0.14	0.11	0.13	0.14	0.053
Indirect bilirubin	0.36	0.23	0.37	0.25	0.102
Alanine transaminase	18.9	17.83	24.7	28.67	0.042
Aspartate transaminase	32.5	27.97	33.75	25.32	0.805
Alkaline phosphatase	108.95	78.37	94.25	47.92	0.003
Total protein	7.44	1.29	7.12	1.5	0.169
Albumin	3.59	0.68	3.49	0.79	0.959
Globulin	3.76	0.88	3.56	1.02	0.118
Albumin/globulin ratio	0.96	0.36	1.03	0.39	0.713
Serum urea	20.45	9.65	24.95	16.22	0.006
Serum creatinine	0.54	0.2	0.68	0.28	<0.001
Serum uric acid	4.3	2.01	4.52	2.79	0.064
Serum calcium	10.34	2.58	10.06	2.52	0.872
Serum phosphorus	5.16	1.09	3.78	0.97	<0.001
Serum sodium	132.9	10.65	132.62	10.94	0.642
Serum potassium	4.25	0.59	4.29	0.61	0.253
Serum chloride	99.24	5.7	99.28	6.67	0.651
Total white blood cell count	14.75	6.03	8.35	6.66	<0.001
Total platelet count	350	155.75	264	181.5	<0.001
Total red blood cell count	4.34	0.97	4.35	0.97	0.551
Total neutrophil count	77.1	14.7	76.05	16.17	0.598
Total lymphocyte count	18.5	13.72	17.55	11.97	0.238
Total monocyte count	3	1.7	3.1	1.42	0.741
Total eosinophil count	0.75	2.2	1	2.22	0.385
Total basophil count	0.2	0.3	0.3	0.32	0.975
Mean platelet volume	11.9	1.8	11.8	1.4	0.903

## Discussion

In this study, a comparison of biochemical and hematological parameters between PTB and EPTB, and adult and pediatric age groups was made. While comparing PTB and EPTB cases, it was observed that the differences between four parameters were statistically significant. The serum calcium levels and total WBC counts in PTB cases were higher, while the levels of serum sodium and total RBC counts were lower in PTB cases when compared to EPTB cases. When analyzing adult and pediatric age groups, the differences between seven parameters were statistically significant. ALT, serum phosphorus, total WBC count, and total platelet counts were higher in the pediatric age group, while ALP, serum urea, and creatinine levels were lower in children.

Serum calcium was found to be elevated in PTB more than in EPTB cases. Various studies have shown that there is hypercalcemia in tuberculosis [9], but a handful of studies also conclude that the levels of serum calcium remain normal in tuberculosis throughout the progression of disease and treatment [10]. Hypercalcemia in TB has been attributed to the over-secretion of extra-renal alpha-1-hydroxylase that is produced by activated macrophages [11]. But it was interesting to note that the median value of calcium was raised only in PTB cases while it was under the normal range in cases of EPTB. One explanation for this finding may be that the alveolar macrophages or dust cells (i.e., the local tissue macrophages of the lungs) are somehow primed to produce more amounts of alpha-1-hydroxylase or maybe that *Mycobacterium tuberculosis* interacts differently with local macrophages of different tissues. Thus, further in-depth studies aiming to understand the biochemical pathways that lead to hypercalcemia in tuberculosis are required. Furthermore, clinicians should pay caution before prescribing vitamin D or calcium supplements to patients with tuberculosis, especially PTB.

The hyponatremia observed in PTB cases can be attributed to the abnormal production of antidiuretic hormone (ADH) in tuberculous lungs [12]. Chronic elevation of ADH is known to aid clinical deterioration in patients with active pulmonary tuberculosis due to its anti-inflammatory action. And thus, suppressing ADH secretion might lessen the disease severity and shorten the time interval required for effective chemotherapy [13]. Therefore, we suggest that routine evaluation of ADH levels along with serum sodium levels should be carried out in all cases of pulmonary tuberculosis, if feasible. 

We observed higher values of serum phosphorus levels, total WBC counts, and total platelet counts in the pediatric age group. Serum phosphorus level tends to be higher with elevated polymorphonuclear leucocyte counts in patients with more severe active disease, suggesting a link to tissue damage [14]. Platelets, through their biological connections with other leukocytes, contribute to matrix metalloproteinase (MMP)-mediated tissue destruction. Increased plasma levels of platelet factor 4; chemokine (C-X-C motif) ligand 4 (PF4; CXCL4), a component of alpha granules specific to platelets, also correlate with disease severity. Numerous observational studies reveal a correlation between platelet count and disease severity and thrombocytosis in TB patients [15]. Thus, based on the above findings, we can hypothesize that children may have been more affected in terms of tissue damage and maybe even disease severity. Moreover, reactive thrombocytosis and raised levels of WBCs and phosphorus may serve as valuable tools indicating active tuberculosis infection and should raise suspicion for severe disease and prompt physicians to initiate counter-measures.

It has also been shown that the lungs contain megakaryocytes, which may have the ability to increase platelet production in response to certain stimuli. And tuberculosis may be one such stimulus leading to the biogenesis of platelets in the lungs [15]. This corroborates our finding that the total platelet counts were higher in cases of pulmonary TB when compared to extrapulmonary TB.

Another set of parameters that showed statistically significant differences between adults and children were serum urea, creatinine, and ALP. These observations may just be due to the inherent differences in physiologies between older and younger individuals and, probably, that may not have been influenced by the presence of tuberculosis. Age is a known factor for variation in kidneys; there is decreased fractional excretion of urea as age advances [16], and serum creatinine concentrations increase steadily with age [17]. Therefore, as expected, both urea and creatinine were higher in adults as compared to children (although within normal ranges). Similarly, due to ongoing bone remodeling and growth, children are known to have higher ALP levels, and as age progresses, the serum levels of ALP decrease; this fact was reflected in our findings as well [18].

The results obtained in this study reveal various differences in hematological and biochemical profiles between PTB and EPTB, and between adult and pediatric cases. These findings suggest that clinicians should keep a lookout for possible complications such as hypercalcemia, hyponatremia, reactive thrombocytosis in PTB cases, and greater tissue damage in pediatric patients. Although these results may not be useful for diagnosing the disease, they have the potential to be quite useful in prognostication. The study will also add to the currently available literature on tuberculosis. Moreover, the significant findings in some of the parameters raise questions about the possible pathophysiology behind them, and thus, further studies are warranted.

This study has certain limitations. The sampling technique used was non-random, and thus, there may have been potential selection bias. As this study was conducted in a tertiary care center where many cases are referred from lower centers, it is likely that more severe cases of TB may have been included, which makes generalization of the results to all cases of TB in the community difficult. Since TB poses such a burden on public health worldwide, we suggest that further studies should be conducted on this topic keeping these limitations in mind.

## Conclusions

We set out to compare the biochemical and hematological profiles in different TB settings and observed that the serum calcium levels and total WBC counts were higher in PTB cases, while the levels of serum sodium and total RBC counts were higher in EPTB cases. ALT, serum phosphorus, total WBC counts, and total platelet counts were higher in the pediatric age group, while ALP, serum urea, and creatinine levels were higher in adults. Increased tissue damage and severity of disease in the pediatric age group, reactive thrombocytosis due to biogenesis in lungs, and abnormal anti-diuretic hormone secretion in PTB cases may be possible explanations for these findings. Thus, the above findings may help clinicians in the early identification of potential complications, and further studies on these parameters should be conducted.
